# The effect of Orem self-care model on the improvement of symptoms and quality of life in patients with diabetes: A scoping review

**DOI:** 10.17533/udea.iee.v42n1e08

**Published:** 2024-04-28

**Authors:** Mohammadamin Jandaghian-Bidgoli, Sheida Jamalnia, Marzieh Pashmforosh, Negin Shaterian, Pouriya Darabiyan, Alireza Rafi

**Affiliations:** 1 Student Research Committee, School of Nursing and Midwifery, Shahid Beheshti University of Medical Sciences, Tehran, Iran. Email: mohammadaminjandaghian@sbmu.ac.ir Shahid Beheshti University of Medical Sciences School of Nursing and Midwifery Shahid Beheshti University of Medical Sciences Tehran Iran mohammadaminjandaghian@sbmu.ac.ir; 2 Department of Nursing and Midwifery, Kazeroun Branch, Islamic Azad University, Kazeroun, Iran. Islamic Azad University Iran; 3 Ph.D student of e-Learning, virtual School, Shiraz University of Medical Sciences, Shiraz, Iran. Email: shjamalnia1988@gmail.com Shiraz University of Medical Sciences virtual School Shiraz University of Medical Sciences Shiraz Iran shjamalnia1988@gmail.com; 4 Behbahan Faculty of Medical Sciences, Behbahan, Iran. Email: marzie_pf@yahoo.com Behbahan Faculty of Medical Sciences Behbahan Iran marzie_pf@yahoo.com; 5 Student Research Committee, Kashan University of Medical Sciences, Kashan, Iran. Email: negin_shaterian@yahoo.com Kashan University of Medical Sciences Student Research Committee Kashan University of Medical Sciences Kashan Iran negin_shaterian@yahoo.com; 6 Student Research Committee, Ahvaz Jundishapur University of Medical Sciences, Ahvaz, Iran. Email: Pouriya.d.k.76@gmail.com Ahvaz Jundishapur University of Medical Sciences Student Research Committee Ahvaz Jundishapur University of Medical Sciences Ahvaz Iran Pouriya.d.k.76@gmail.com; 7 M.Sc of Nursing, Behbahan Faculty of Medical Sciences, Behbahan, Iran Email: alirezarafi72@gmail.com‏. (Corresponding author) Behbahan Faculty of Medical Sciences Behbahan Faculty of Medical Sciences Behbahan Iran alirezarafi72@gmail.com

**Keywords:** Diabetes Mellitus, models, nursing, self-care, quality of life., Diabetes Mellitus, modelos de enfermería, calidad de vida, autocuidado., Diabetes Mellitus, modelos de enfermagem, qualidade de vida, autocuidado.

## Abstract

**Objective:**

to evaluate the association of Orem self-care model improvement of symptoms and quality of life in patients with diabetes.

**Methods.:**

A scoping review was carried on bibliographic databases: PubMed-Medline, Scopus, SID and Magiran. The inclusion criteria encompassed studies examining the impact of the Orem self-care model on diabetic patients. Studies considered for inclusion needed to have full-text availability and be written in either English or Persian, with key words including “Models”, “Nursing”, “Quality of Life”, and “Diabetes Mellitus”. CONSORT checklist and STROBE statement were selected for quality assessment.

**Results.:**

A total of 9 studies were included, all using quantitative methodology and focusing on adults or older adults. The majority of articles focused on quality of life and diabetic symptoms. 8 studies showed positive outcomes after implementation of the model. The findings indicate that this model led to an enhanced level of self-efficacy, improved quality of life, and better self-care practices among diabetic patients.

**Conclusion.:**

Orem self-care model can reduce the diabetic symptoms and improve the quality of life, self-efficacy and self-care in these patients.

## Resumen

**Objetivo.:**

Evaluar la asociación del modelo de autocuidado de Orem en el mejoramiento de los síntomas y en la calidad de vida en pacientes con diabetes.

**Método.:**

Se realizó una revisión de alcance empleando las bases bibliográficas PubMed-Medline, Scopus, SID y Magiran. Los criterios de inclusión abarcaron estudios que examinaran el impacto del modelo de autocuidado de Orem en pacientes diabéticos. Los estudios considerados para su inclusión debían tener disponibilidad de texto completo y estar escritos en inglés o persa, con palabras clave como: “Models”, “Nursing”, “Quality of Life” y “Diabetes Mellitus”. Se utilizaron para la evaluación de la calidad de los estudios la lista de comprobación CONSORT y la declaración STROBE.

**Resultados.:**

Se incluyeron un total de 9 estudios, todos ellos con metodología cuantitativa y centrados en adultos y en ancianos. La mayoría de los artículos se estudiaron la calidad de vida y los síntomas diabéticos. 8 estudios mostraron resultados positivos tras la aplicación del modelo de Orem. Los hallazgos indican que este modelo condujo a un mayor nivel de autoeficacia, mejor calidad de vida y mejores prácticas de autocuidado entre los pacientes diabéticos.

**Conclusión:**

. El modelo de autocuidado de Orem puede ayudar a disminuir los síntomas diabéticos y mejorar la calidad de vida, la autoeficacia y el autocuidado en estos pacientes

## Resumo

**Objetivo.:**

Avaliar a associação do modelo de autocuidado de Orem na melhora dos sintomas e na qualidade de vida de pacientes com diabetes.

**Métodos.:**

Foi realizada uma revisão de escopo usando os bancos de dados PubMed-Medline, Scopus, SID e Magiran. Os critérios de inclusão incluíram estudos que examinaram o impacto do modelo de autocuidado de Orem em pacientes diabéticos. Os estudos considerados para inclusão tinham que estar disponíveis em texto completo e escritos em inglês ou persa, com palavras-chave como: "Models", "Nursing", "Quality of Life" e "Diabetes Mellitus". A lista de verificação CONSORT e a declaração STROBE foram usadas para avaliar a qualidade dos estudos.

**Resultados.:**

Foram incluídos 9 estudos, todos com metodologia quantitativa e com foco em adultos e idosos. A maioria dos artigos estudou a qualidade de vida e os sintomas diabéticos. Oito estudos mostraram resultados positivos após a aplicação do modelo de Orem. Os achados indicam que esse modelo levou a um nível mais alto de autoeficácia, melhor qualidade de vida e melhores práticas de autocuidado entre os pacientes diabéticos.

**Conclusão.:**

O modelo de autocuidado de Orem pode ajudar a diminuir os sintomas da diabetes e melhorar a qualidade de vida, a autoeficácia e o autocuidado desses pacientes.

## Introduction

Diabetes originates from impairment in the metabolism of proteins, carbohydrates and lipids.^1^ It is a chronic metabolic disorder that is defined and recognized by the increase in blood sugar caused by defects in the secretion or action of insulin or both. This disease leads to many complications such as limb amputation, cardiovascular problems, kidney diseases, blindness, and long-term disabilities.^2^ Diabetes is one of the most common non-communicable diseases that has the highest prevalence rate among metabolic diseases. More than 90% of diabetic patients suffer from type 2 diabetes. This disease is associated with short-term and long-term complications that are irreversible in many cases.^3^ Complications and deaths caused by diabetes are among health problems all over the world. The increase in the number of patients with type 2 diabetes indicates a global epidemic.^4^ In addition to direct complications, diabetes also has many costs that affect the lives of the patients. In 2017, 327 billion dollars was the total cost of diagnosed diabetes.^5^. 

The prevalence of type 1 and type 2 diabetes is increasing worldwide, but the rate of type 2 diabetes is higher than that of type 1.^6^ Diabetes is a chronic condition that imposes major threats to the mankind and affected 435 million individuals in 2017^7^. It will involve 552 and 624 million patients by 2030 and 2040, respectively.^8^^,^^9^ According to the International Diabetes Federation (IDF), Iran ranks third among Middle Eastern countries in terms of diabetes (5.4 million cases). It is expected that the rate of diabetes will reach 9.2 million Iranians by 2030.^10^ Aging population will be another effective factor in increasing the prevalence of diabetes in the future.^11^ The increase in the number of diabetic patients has faced the care provider organizations with increasing financial problems and a decrease in the number of care providers.^12^ 15-20% of hospital beds are occupied by patients with diabetes and it is difficult to determine which of these patients require more medical attention.^13^ Regardless of age, country and economic conditions, diabetes is a major health challenge and has a global impact, so that the prevalence of diabetes is reaching an alarming level.^14^ Therefore, the prevention and control of this disease is considered one of the health challenges, so that one of the goals of the Ministry of Health, Treatment and Medical Training of Iran is to stop the spread and growth of diabetes until 2025.^15^

One of the most important clinical goals of nursing care is to reduce the severity of disease symptoms and the stress caused by it in order to improve the quality of life in these patients. Without patient participation, favorable outcomes cannot be considered. Quality of life is a crucial aspect of healthcare, particularly in the context of nursing care. The primary clinical objective for nurses is to alleviate the severity of disease symptoms and mitigate the associated stress experienced by patients. By doing so, the overarching goal is to enhance the overall quality of life for individuals undergoing medical treatment.^16^ Self-care behaviors are one of the most important components which requires special attention. These behaviors in diabetic patients includes appropriate glycemic control, following a healthy diet, taking medicines regularly, monitoring blood sugar, taking care of feet and doing sports activities.^17^ Self-care behaviors are naturally multidimensional and include all the activities focused on health maintenance, disease prevention and treatment. These kinds of behaviors are done purposefully and with the consent of the patient.^18^ Improving the self-care behaviors is the first step toward helping the diabetic patients. Normally, self-care practices can reduce the costs and prevent acute or chronic complications of diabetes.^19^^,^^20^ Appropriate behaviors can lead to a decreased level of cardiovascular risks among diabetic patients.^21^ Since self-care activities are effective in glycemic control and other outcomes in diabetic patients, it can be claimed that self-care behaviors can be an underlying reason for mortality among them.^(22, 23)^

One of the models that can contribute to the patient's self-care is Orem self-care model.^24^ According to Orem, humans have the ability to take care of themselves, and if this ability is disturbed, nurses help them to regain this ability.^25^ Understanding the impact of the Orem Self-Care Model on symptom alleviation and quality of life is crucial for healthcare professionals and policymakers. Positive findings could lead to the development of targeted interventions and strategies that enhance patient outcomes, potentially improving the overall quality of care for individuals with diabetes. The need for research on factors affecting self-care based on the needs of patients to increase their self-care ability seems necessary. Hence, this study aimed to evaluate the association of Orem self-care model improvement of symptoms and quality of life in patients with diabetes. 

## Methods

This scoping review adheres to PRISMA guidelines. The review was registered on PROSPERO (ID:CRD42022367809). (ID:CRD42022367809) Also, this research has been approved by the ethics committee of Behbahan Faculty of Medical Sciences (code of ethics: IR.BHN.REC.1402.002)

*Search strategy.* The following databases were searched: PubMed, Scopus, SID, Magiran. Articles published between 2011 and October 31, 2022 were included in the search strategy. The search included following terms: 1- Orem Self-Care Model (Text Word) OR Model, Orem Self-Care (Mesh Term) OR Self-Care Model, Orem (Mesh Term) OR Orem Self Care Model (Mesh Term); 2- Diabetes Mellitus (Text Word) OR Diabetes Mellitus, Noninsulin-Dependent (Mesh Term) OR Diabetes Mellitus, Ketosis-Resistant (Mesh Term) OR Stable Diabetes Mellitus (Mesh Term) OR Type 2 Diabetes Mellitus (Mesh Term) OR Noninsulin Dependent Diabetes Mellitus (Mesh Term) OR NIDDM (Mesh Term) OR Adult-Onset Diabetes Mellitus (Mesh Term) OR IDDM (Mesh Term) OR Type 1 Diabetes OR Autoimmune Diabetes (Mesh Term) OR Juvenile-Onset Diabetes (Mesh Term) OR Sudden-Onset Diabetes Mellitus; and 3- 1 AND 2. In order to minimize the number of missing data, Google Scholar was also searched. The search strategy was completed by snowball search (e.g., existing systematic reviews and included articles) of included studies.

*Inclusion/Exclusion Criteria.* The inclusion criteria were as following: Studies published in English or Persian with full text available, participants aged 10-65 years, participants with the diagnosis of diabetes mellitus at any stage of the disease. Included interventions were those that aimed to implement Orem model. Moreover, the intervention can be emanated from either inpatient or outpatient setting. Additionally, randomization may reduce the risk of bias, it may not be fully possible in health care. Hence, the studies with nonrandomized or one-group study designs were included. 

The exclusion criteria were as following: interventions of models other than Orem model; intervention specifically aimed to treat homeless patients, participants with physical or mental handicap and patients involved in forensic process. Gray literature which did not publish as articles, such as posters, organizational projects, class presentations, health messages and other similar literature did not meet the eligibility criteria. 

*Quality Assessment.* For the assessment of observational studies, STROBE statement (Strengthening the Reporting of Observational studies in Epidemiology) was used. The statement consists of 22 items (26). In addition, CONSORT checklist was used for the assessment of interventional studies ^27^.

*Data Extraction.* In order to find eligible studies, titles and abstracts from databases and additional sources were assessed by two authors independently. Then, the retrieved full texts were checked by the same two authors. Any disagreement was resolved by consultation with the third author. Unavailable data was requested from study authors. 

Two authors were responsible for data extraction of included studies. The following items were extracted: 1- General information (Author, publication year, reference, study type, mean age, sample size); 2- Participants; 3- Measurement tool; 4- Intervention period, 4- Control; 5- Outcome; 6- Main finding or key points.

**Figure 1:**
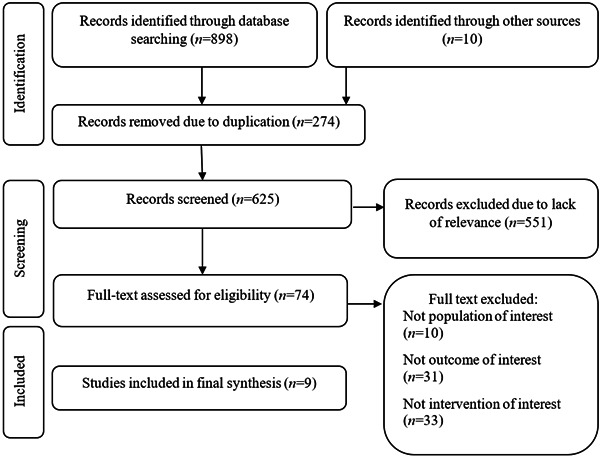
PRISMA Flowchart of selected studies

## Results

The initial search yielded 259 records on PubMed, 226 on Scopus, 215 on SID and 189 on Magiran. After removal of duplicates, the final records were 625. By reviewing the titles, the eligible articles reduced to 321. The selection by abstract brought the total to 74. The main purpose of the study was to select the studies have examined the special association between Orem self-care model and diabetic patients. This led to a significant reduction of eligible articles. Eventually, 9 studies met the inclusion criteria and were considered. Figure 1 shows the process of study selection. 

Two studies were from PubMed, whilst 6 were extracted from SID and Magiran. Additionally, one study gained from snowball method. Almost, all of the studies were interventional. There were 597 participants in this systematic review. 

Within the selected studies, there were some items investigated: 3 articles focus on diabetes-related psychical symptoms,^28^^,^^29^ 3 articles focus on self-efficacy,^30^ 2 articles focus on quality of life,^31^^-^^33^ 1 articles focus on self-care.^34^^-^^36^ 8 out of 9 articles showed a positive statistically significant correlation between Orem self-care model and positive outcomes in diabetic patients. The details and main findings are given in Table 1. For instance, it was found that implementing Orem's self-care model can lead to a decreased level of diabetic neuropathy.^28^ As well, the study showed that the group receiving Orem's model has more appropriate level of self-care.^29^ Similarly, as previously mentioned, other factors such as quality of life^31^^-^^33^ and self-efficacy^30^ had improved in the group receiving Orem's self-care model. 

An overview of quality assessment of studies using STROBE statement and CONSORT checklist and is presented in Tables 1. For the quality assessment of observational studies, the Strengthening the Reporting of Observational Studies in Epidemiology (STROBE) was used. STROBE consists of a checklist with 22 items. The total scores lower than 17 were indicative of low methodological quality. ^37^. On the other hand, experimental and quasi-experimental studies were assessed by the CONSORT checklist. This checklist includes 25 items (38). Two authors were responsible for methodological quality of included studies. The third author resolved any disagreement between the reviewers. Seemingly, the included studies possessed acceptable quality. 

**Table 1:** CONSORT and STROBE checklist scores of selected studies

Author and year of publication	Reference	Type of study	Score
Ahrary *et al.* (2020)	29	Randomized controlled trial	20/24*
Hemmati Maslakpak *et al.* (2017)	30	Controlled trial	21/24*
Mansouri *et al.* (2017)	31	Controlled trial	22/24*
Baraz *et al.* (2017)	32	Controlled trial	22/24*
Borji *et al.* (2017)	33	Controlled trial	21/24*
Shahbaz *et al.* (2016)	35	Controlled trial	20/24*
Ganjlo *et al.* (2015)	34	Randomized controlled trial	21/24*
Ghafourifard *et al.* (2015)	36	Controlled trial	22/24*
Khosravan *et al.* (2015)	37	Descriptive	20/22^†^

*: CONSORT score; †: STROBE score

## Discussion

The results indicated Orem self-care model can lead to an improved level of self-efficacy, quality of life and self-care among the diabetic patients Also, the diabetic symptoms diminished. In this way, similar studies have showed that self-care behaviors can lead to the improvement of quality of life and reduction of costs.^39^ It was proved that Orem self-care model is effective in changing the dietary habits among the participants,^35^ as other studies have shown the efficacy of educational programs for improving the dietary behaviors and controlling the blood sugar are effective.^40^ However, the seasonal preferences need to be taken into account. For instance, in autumn, the desire for protein and fat increases, whilst patients in summer prefer to consume more carbohydrate.^41^ Even, religious beliefs, culture and lifestyle can affect dietary habits. In other words, dietary habits are individualized.^42^ Even though, culture can alter the perception of patients regarding healthcare providers.^43^

Some of the included studies were conducted on patients with diabetic foot ulcer and showed promising results.^29^^,^^34^ Other similar programs conducted in Iran were indicative of effectiveness of self-care training programs among diabetic patients as they could be helpful in wound healing process.^44^ Similarly, studies in other countries highlighted the pivotal role of educating patients for the self-care.^45^ It is proved that controlling the diabetic foot ulcer mainly depends on the patient.^46^ However, the factors such as marital status, educational level, gender, age and occupation paly important role in the level of self-care.^47^ For instance, it is claimed that rate of self-care is higher in females compared with males.^48^ Likewise, single patients pay less attention to their self-care, especially regarding diabetic foot ulcer.^48^ It is because stronger emotional and social bonds exist among married patients.^49^

The study participants were middle-aged and older adults. In this way, a study on older adults with diabetes showed health-related quality of life was moderate ^50^. Though, married older adults had higher scores of quality of life ^51^. Education was another multi-dimensional factor which can lead to higher contribution in the areas such economy, society and politics. Educational level directly affects the quality of life in the diabetic patients ^52^. On the other hand, illiterate individuals are less familiar with coping strategies. Also, these individuals are in an inappropriate economic situation, which can cause more stressful situation and lower level of quality of life ^53^. 

The results indicated that Orem self-care model in female patients with diabetes suffering from neuropathy can be effective in reeducation of symptoms and severity and it led in improvement of knowledge, attitude and other related skills.^28^^,^^36^ However, their performance and self-care abilities was reported moderate.^36^ Other studies conducted in Iran have also shown that training can be effective in communication skills and self-control in patients with diabetic neuropathy.^54^ In addition, a study in USA proved that education is effective in regulation of cognitive and emotional states.^55^

The psychological effect of self-care programs is also undeniable. For instance, a study showed that self-management were effective in improvement of mood condition in diabetic patients.^56^ However, many programs do not consider education in the area of psychological skills, as problems such as depression are common in diabetic patients.^57^ A study in Iran showed psychological training can alter the level of stress, anxiety and depression among diabetic patients.^58^ Additionally, existential need in patients encourages them to seek meaning in life and this can help to increase their well-being.^59^ In explaining the role of the spiritual dimension of patients, it can be acknowledged that patients who receive more services have more ability to deal with psychological pressure and psychological trauma.^60^ For the success in implementing such programs, the imperative role of economy should not be neglected. Economic well-being and availability of financial services can be effective in medical adherence of patients.^61^ Naturally, unemployed individuals or those with a low-income profession are more vulnerable to experiencing adverse effects of the disorders.^62^

Additionally, comorbidities play an important part in lack of self-care behaviors in diabetic patients.^63^ Obstacles such as physical limitations, polypharmacy, adverse symptoms and lack of social and emotional support exist that can have negative impact on self-control and self-care behaviors.^64^ However, it was claimed that increasing comorbidities result in poor diabetes self-care.^65^

One of the findings was focused on gestational diabetes, which indicated Orem self-care model can be effective for the patients.^30^ Similarly, other studies also proved that self-care training can bring about positive outcome both in Iran and other countries.^66^^,^^67^ However, one study claimed that education cannot alter the scores related to blood sugar.^68^ Specially, after COVID-19 pandemic, the psychological problems have increased, which it may be reduced in gestational diabetes by self-care training.^66^^,^^69^ Notably, lack of knowledge regarding health is a risk factor for poor self-management in women suffering from gestational diabetes.^70^ Likewise, It is believed that lack of social support is another important risk factor for women and has adverse effects on gestational outcomes.^71^ One of the important steps toward coping behaviors in gestational diabetes is acceptance. Emotional stability is a factor which may lead to the acceptance.^72^

In conclusion, looking at the relation found between Orem self-care model and diabetic patients' outcome, it can be stated the model can be effective for the diabetic patients. Positive and supportive implementation of the model can improve patients' quality of life, self-efficacy, and self-care behaviors and reduce diabetic symptoms. 

The studies analyzed in this review have revealed that Orem self-care has a significant association with positive outcomes among diabetic patients. This shows that Orem self-care model can induce transformational alterations in self-care and quality of life in diabetic patients. However, future studies need to focus on other outcomes related to diabetes as it is a multi-dimensional disease. Specially, psychological outcomes need to be considered. 

Limitations. Despite the efforts to present a perfect systematic review, there were some shortcomings. In practice, the studies were included if were published in English or Persian owing to inclusion criteria. Also, the full text of one article was not available. Additionally, there were various tools for the measurement of outcomes. Although the search strategy was designed precisely and snowball search of included studies was considered, there may be some missing data. Absolutely, there is need for conducting more studies to complement the findings of current study. 
